# Sensory, Emotional and Cognitive Contributions to Anxiety in Autism Spectrum Disorders

**DOI:** 10.3389/fnhum.2017.00020

**Published:** 2017-01-24

**Authors:** Mikle South, Jacqui Rodgers

**Affiliations:** ^1^Departments of Psychology and Neuroscience, Brigham Young UniversityProvo, UT, USA; ^2^Institute of Neuroscience, Newcastle UniversityNewcastle, UK

**Keywords:** autism spectrum disorder, anxiety, medial prefrontal cortex, intolerance of uncertainty, alexithymia, mindfulness, sensory function

## Abstract

Severe symptoms of anxiety add substantial additional burden to many individuals diagnosed with Autism Spectrum Disorder (ASD). Improved understanding of specific factors that contribute to anxiety in ASD can aid research regarding the causes of autism and also provide targets for more effective intervention. This mini-review article focuses on emerging evidence for three concepts that appear to be related to each other and which also strongly predict anxiety in ASD samples. *Atypical sensory function* is included in the diagnostic criteria for ASD and is likely an important contributor to anxiety. Difficulties in understanding and labeling emotions (*alexithymia*), although a co-morbidity, may arise in part from atypical sensory function and can lead to confusion and uncertainty about how to respond to social and emotional situations. *Intolerance of uncertainty* *(IU)* describes people who have a particularly hard time with ambiguity and is known to be a key mechanism underlying some anxiety disorders. While evidence for linking these ideas is to date incomplete, we put forward a model including each concept as a framework for future studies. Specifically, we propose that IU is a critical mediator for anxiety in ASD, and explore the relationships between sensory function, alexithymia and IU. We further explore the role of the medial prefrontal cortex (mPFC) in regulating emotional response, in connection with limbic and insula-based networks, and suggest that disrupted integration in these networks underlies difficulties with habituation to strong emotional stimuli, which results in an enhanced perception of threat in many people with ASD. Behavioral and biologically-based treatments for anxiety in ASD will benefit from attending to these specific mechanisms as adjunct to traditional interventions.

## Introduction: Anxiety in Autism Spectrum Disorders

Severe symptoms of anxiety co-occur frequently in autism spectrum disorder (ASD; Kerns and Kendall, 2014). Kerns et al. (2014) identified several aspects of anxious presentation in ASD that are similar to those found in standard diagnostic systems, but also a number of atypical expressions that seem specific to ASD, including social discomfort not associated with a fear of negative evaluation; compulsive behavior that does not seem motivated by distress relief, and highly unusual phobias. Improved understanding of the cognitive and emotional mechanisms which underlie anxiety in ASD may provide insight regarding the neurobiology of both conditions and create more specific targets for biological and behavioral intervention (Hashemi et al., 2016; Kerns et al., 2016; Rodgers et al., 2016b).

### Targeting Specificity

This review examines three ideas that may have particular salience for understanding the development and maintenance of anxiety in ASD: (1) atypical sensory function, which is included in the diagnostic criteria for ASD; (2) difficulty identifying/labeling emotions (alexithymia), which has been shown to be frequently severe in ASD; and (3) intolerance of uncertainty (IU), which has been suggested as a critical pathway to anxiety in ASD. While these ideas come from very different backgrounds, existing research shows that they are closely related, at least as measured with current instruments. A critical challenge is to define how these ideas diverge biologically and behaviorally.

## Atypical Sensory Function and Anxiety in ASD

Sensory function in ASD samples may be marked by both over-responsivity (e.g., experiencing various sounds as painful) and under-responsivity (e.g., repeated touching of objects). Both extremes may occur in the same children (Green and Ben-Sasson, 2010; Wigham et al., 2015; Watts et al., 2016). Green and Ben-Sasson (2010) proposed separate causal models for sensory over-responsivity and anxiety in ASD that moves in both directions. This seminal article suggested three guidelines for future studies: (1) using questionnaires and also psychophysiological challenge; (2) the need for prospective/longitudinal studies and also intervention studies; and (3) the development of animal studies.

Several aspects of the Green and Ben-Sasson (2010) framework are finding empirical support. Rodgers et al. (2016b) collected data from parent focus groups and factor analysis to modify an existing anxiety questionnaire in pursuit of increased relevance for both clinical and research use with ASD children. Sensory-based anxiety is a critical subscale along with subscales for difficulty with uncertainty and performance anxiety. Sensory concerns appear to be more prevalent in ASD samples than in control groups with developmental concerns, including special educational needs (Green et al., 2016) and adults with intellectual disabilities (Gillott and Standen, 2007). In ASD samples, severity of anxiety appears to be higher in individuals with more severe sensory dysfunction (Gillott and Standen, 2007; Uljarević et al., 2016).

There are now a few studies using psychophysiological methods to study sensory function. Green et al. (2013, 2015) reported two fMRI studies in high-functioning ASD youth during a challenge of mildly aversive sensory stimuli. In general, ASD participants showed more activation than controls in primary sensory areas, amygdala and orbitofrontal cortex in response to auditory stimuli. This activation was correlated with parent-reported anxiety and also with sensory over-responsiveness beyond the association with anxiety. Brain activity in the ASD samples was especially heightened when multiple sensory modalities (auditory and tactile) appeared simultaneously. The authors highlighted difficulties with habituation as a key underlying feature of this overresponsiveness. Corbett et al. (2016) reported that cortisol response to stress was higher for ASD children than controls during an ecologically-relevant peer interaction. Greater sensory dysfunction was associated with increased stress, and diagnosis was a significant moderator of the relationship between sensory function and stress response.

Animal researchers likewise have developed increased awareness of the importance of sensory function as a dependent variable (Kas et al., 2014). Adult mice whose whiskers had been trimmed at 10 days old (which causes pronounced tactile deficits) exhibited both social deficits and profound changes in amygdala activation, including hypersensitivity to stress (Soumiya et al., 2016).

## Understanding Emotions: Alexithymia and Mindfulness in ASD

*Alexithymia* is a term used to characterize difficulties with emotional awareness, especially referring difficulty in identifying and describing internal emotional states (Cameron et al., 2014). Alexithymia cuts across diagnostic boundaries including associations with anxiety, depression and autism (Grabe et al., 2004; Mennin and Fresco, 2009; Bird and Cook, 2013). Whereas alexithymia is generally thought of as a psychological trait, *psychological mindfulness* is a term used to describe the skill to attend to one’s experience in the present moment in a non-judgmental way (Kabat-Zinn, 2015). Our previous work (Maisel et al., 2016) has highlighted the contribution of emotional acceptance, which describes the ability to allow one’s internal experience to be as it is and not to push feelings away, to anxiety in ASD. Decreased emotional awareness associated with alexithymia may impair the ability to develop emotional acceptance.

Some cognitive and emotional difficulties frequently seen in autism may be more related to alexithymia than to core autism symptoms (Caria et al., 2011; Allen et al., 2013; Bird and Cook, 2013). For example, Bird et al. (2011) reported that overall, a small sample of ASD adults showed less attention to faces compared to controls; importantly, the balance of time spent looking at the mouth rather than the eyes was predicted by alexithymia scores and not autism severity. An fMRI study from the same group (Bird et al., 2010) reported that alexithymia predicts brain response to empathy-generating situations in both ASD and control groups and that alexithymia, not autism symptoms, seems to account for between-group differences in empathy symptoms.

Maisel et al. (2016) reviewed symptom questionnaires completed by adults diagnosed with ASD (*n* = 76) and neurotypical controls (*n* = 75), showing strong correlations between dimensional measures of autism symptoms and several measures of anxiety. Structural equation modeling demonstrated that this relationship was almost completely accounted for by a combination factor of alexithymia, emotional acceptance and IU. High alexithymia levels are also seen in young children diagnosed with ASD (Griffin et al., 2015).

## Intolerance of Uncertainty in ASD

*IU* is a cognitive construct referring to decreased thresholds for the perception of ambiguity and enhanced discomfort with ambiguity (Dugas et al., 1998). The negative affect associated with IU is seen in generalized anxiety disorder but also other anxiety disorders, depression and autism (McEvoy and Mahoney, 2012; Einstein, 2014).

Boulter et al. (2014) reported on the relationship between IU and anxiety in ASD using both parent and child reports on the Intolerance of Uncertainty Scale for Children (IUS-C) and Spence Children’s Anxiety Scale (SCAS). They found significantly higher IUS-C and SCAS scores for ASD children and adolescents than a typical comparison group, a finding recently replicated by Neil et al. (2016) with similarly large samples. Importantly, Boulter et al. (2014) report a “causal mediational model” in which IU almost completely mediated the relationship between diagnostic group and anxiety scores.

Wigham et al. (2015) reported that IUS-C and SCAS-P anxiety scores were significant mediators of the relationship between sensory function and core symptoms of repetitive/restricted behaviors in ASD children, with somewhat different pathways associating under- or over-responsive sensory function with category of repetitive behavior (motor behaviors or sameness behaviors). This was also replicated and extended by Neil et al. (2016), who included a large group of typically developing children. Parent-report scores were higher for the ASD than control group for sensory sensitivities, IU and anxiety scales. Hierarchical regression analysis indicated that IU significantly predicted sensory sensitivity in both ASD and typical groups, but the predictive power of IU was much greater in the ASD group. Links between sensory sensitivity, IU and anxiety have also been reported in mothers of ASD children (Uljarević et al., 2015).

Hodgson et al. (2016) conducted focus groups of nine parents of ASD children without intellectual disabilities. The parents easily differentiated IU from a general dislike of change, including discomfort with unknown situations such as delayed flights on a planned trip; and discomfort with uncertainty even in known situations, when a child moves to middle school and is required to use a pen instead of a pencil but is afraid she might make a mistake and not be able to erase it. Keefer et al. (2016) reported that ASD children completing the *Face Your Fears* cognitive behavioral therapy (CBT) protocol had significantly reduced parent-reported anxiety symptoms. Importantly, pre-treatment IU scores significantly mediated the change in anxiety scores such that children with higher pre-treatment IU showed less improvement of their anxiety symptoms.

## Building Models

**Figure 1:**
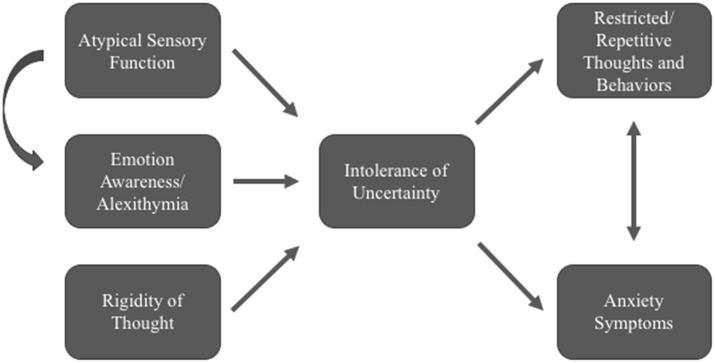
**Exploratory model of possible pathways related to Intolerance of uncertainty (IU) and anxiety in Autism Spectrum Disorder (ASD).** Evidence is growing for IU as a significant mediator of anxiety in ASD samples.

We have presented evidence for associations between anxiety in ASD with atypical sensory function, emotion recognition and regulation (trait alexithymia and mindfulness skills), and cognitive (IU) difficulties. While these represent just a few among many possible pathways to emotion dysregulation in autism (Mazefsky et al., 2013; White et al., 2014), recent clinical- and measurement-based research studies have highlighted the relevance of each of these ideas for future study. We propose the following exploratory model as a framework for future studies to develop areas of overlap and causal links among these constructs (see Figure 1). For example, atypical sensory functioning may exacerbate uncertainty for both external and internal (interoceptive) stimuli. Our model includes related ideas that have not yet received adequate research attention but may also provide explanatory power. For example, cognitive and behavioral rigidity may create enhanced uncertainty about whether exact rules will be followed, and may also be associated with repetitive and restricted behaviors (see e.g., Wigham et al., 2015; Neil et al., 2016).

A major difficulty for many of the studies reviewed here is how to separate the underlying theoretical mechanisms from each other. While this model arose from clinical observation, other factors such as methods bias (e.g., questionnaires completed by the same raters) make it difficult to tease them apart using only survey data. It is likewise difficult to understand relationships between behavioral data and psychophysiological data (Geurts et al., 2009; Watts et al., 2016).

There is an urgent need for experimental and ecologically-valid paradigms to address these questions. One question is how valid are existing measures of these constructs for autism samples. This has so far been taken up in the autism and anxiety literature, mostly for pediatric samples. Several studies suggest some relevance for existing measures but also recognize autism-unique aspects of anxiety that require additional attention (Kerns et al., 2014; Rodgers et al., 2016b). Standard measures for alexithymia include the Toronto Alexithymia Scale-20 (Bagby et al., 1994) and the Bermond-Vorst Alexithymia Questionnaire (Vorst and Bermond, 2001). Mindfulness is most frequently measured using the Five Facet Mindfulness Questionnaire (Baer et al., 2006) though there are many others. IU is captured using the IU Scale, often the short, 12-item version (Carleton et al., 2007). While these measures demonstrated more-than-typical levels of each construct in a self-reporting sample of ASD adults (Maisel et al., 2016), formal studies of validity and factor structure have not been undertaken in autism populations. Such studies are clearly necessary for forwarding work in this field.

## Autism, Anxiety and Integration of mPFC

Shalom (2009) and others have highlighted the potential contributions of disrupted integration in medial prefrontal cortex (mPFC) function across a variety of abilities in ASD, including memory and visual processing (Lecouvey et al., 2015; Smith et al., 2015; Tzur, 2015). To date, however, there has been little published research specifically related to the mechanisms that link mPFC, anxiety and autism symptoms (though see possible relationships in rodent models, Truitt et al., 2007).

Fear and anxiety critically depend on medial aspects of prefrontal cortex including mPFC and anterior cingulate cortex (ACC), with key connections to amygdala, hippocampus and insula regions among others (Giustino and Maren, 2015). Essentially, it is thought that prefrontal cortex functions to provide top-down regulation of emotional response to incoming signals, and disruption in this network leads to difficulty managing that emotional response and the subsequent development of maladaptive emotional states including excessive anxiety. For example, Herry et al. (2007) found that unpredictability in sequences of sound pulses was associated with anxiety-like behavior in both mice and humans, with enhanced/sustained amygdala activity in both species. The authors conclude (p. 5964) that “habituation of neuronal activity in the amygdala represents an evolutionary conserved mechanism by which temporal predictability of perceptual input leads to adaptive changes in emotional behavior”. That is, difficulties with uncertainty at a primitive level (the amygdala) seem to be related to difficulties in flexible emotional response.

Several studies of mPFC dysfunction in ASD samples have focused on social disabilities such as face processing (Kleinhans et al., 2009; Swartz et al., 2013) conclude that decreased regulation of amygdala by ventromedial PFC (vmPFC) underlies reduced habituation of amygdala in ASD, resulting in decreased modulation of response to social stimuli. This same reasoning applies just as well to explanations for anxiety: reduced modulation of limbic activity via higher cortical systems may result in chronic, unchecked perception of threat and subsequent anxiety (Amaral et al., 2003). Top et al. (2016) conducted an fMRI study of fear conditioning and extinction in adults with ASD, concluding that the ASD group failed to recognize safety cues, and thus were delayed in responding to changes between threat to safety contexts.

One key question is how much these difficulties with adaptation/flexibility arise from bottom-up or top-down integration (Shalom, 2009). For example, experience with ambiguity may be increased in ASD through multiple channels. Computational models created by Pellicano and others (Pellicano and Burr, 2012; Lawson et al., 2014; Sinha et al., 2014; Van de Cruys et al., 2014) suggest that people with ASD are unable to effectively modulate current experience with information from prior experience, leading to an overwhelming experience for new information. While Pellicano and Burr (2012) have mostly focused this account on sensory and perceptual stimuli, others have noted the relevance of such a model for explaining other aspects of autism including social difficulties based on more top-down approaches (Brock, 2012; Sinha et al., 2014). Disrupted connectivity between other regions of mPFC (including ACC) and the limbic system (including hippocampus) may also affect emotion regulation and anxiety.

Insula cortex is critical for integration of interoceptive and sensory information and regulating emotional response (Gasquoine, 2014). Alexithymia and empathy are associated with insula activity in ASD and controls (Bird et al., 2010). A seminal article on the neurobiology of IU (Grupe and Nitschke, 2013) suggests that medial orbitofrontal cortex and insula provide information about the subjective value of potential events, balancing an individual’s feelings of probable risk separate from a reasoned consideration of risk. Disruptions to this network may then lead to “more vivid or visceral simulations of potential events” (p. 492) and heighten a bias towards threat especially in uncertain situations. Future research should continue to examine the interplay between specific aspects of reduced mPFC integration in ASD.

## Implications for Treatment

Traditional CBT approaches may focus on mechanisms of anxiety that are less relevant for ASD youth and adults (Kerns et al., 2014). Rapidly emerging data suggests that standard behavioral and pharmacological mental health interventions will benefit from understanding and targeting highly-specific underlying mechanisms (Keefer et al., 2016; Walsh et al., 2016). For example, interventions directed at improving sensory integration could potentially reduce anxiety in ASD, although little research in this area has been done to date (Watts et al., 2016). Mindfulness based treatments (MBT)—aimed at increasing emotional awareness as well as the ability to cope with strong emotions–have been adapted for ASD in several trials with encouraging success in reducing anxiety and increasing positive affect (Spek et al., 2013; Dykens et al., 2014; Jones et al., 2014; de Bruin et al., 2015). Difficulties with habituation and flexibility, associated with disrupted integration between mPFC and limbic regions, can also be treated directly with benefits for anxiety and depression (Kenworthy et al., 2014; Lawson et al., 2015; Wallace et al., 2016). IU is a critical target for future research in both causes and treatment for anxiety in some individuals diagnosed with ASD. Keefer et al. (2016) suggest that directly targeting IU in ASD may be a helpful adjunct to usual CBT approaches. Rodgers et al. (2016a) developed a manualized, parent group based treatment program for young people with ASD which focused specifically on IU, which preliminary evaluation indicates has promise as a treatment option for young people with ASD and anxiety.

Individual differences provide essential context for any treatment implications. For instance, we propose here that difficulties with habituation contribute to atypical sensory function in ASD. However, Schoen et al. (2008) identified two habituation subgroups within a sample of 40 children with ASD, one characterized by slower latency and faster habituation and the other by faster latency and slower habituation in response to sensory stimuli. Similar findings have been reported by Hirstein et al. (2001). It may be that the link between sensory function and anxiety holds only for those with difficulty habituating to new stimuli. As noted by Grupe and Nitschke (2013), limited habituation in mPFC biases information processing towards negative/threatening frameworks; individuals for whom this system is working more typically may be less susceptible to anxiety even if they are diagnosed with ASD.

## Summary

There is an urgent need to investigate the relevance of many possible underlying mechanisms related to emotion dysregulation in ASD. This mini-review article has focused on three such mechanisms—atypical sensory function, alexithymia and IU–that appear to be closely related to each other and that strongly predict anxiety in ASD. Although support for each of these concepts is emerging, evidence linking the three constructs together remains incomplete. Thus, this model is speculative, but we believe it builds a helpful framework for future studies. Such difficulty integrating cognitive and emotional information putatively arises at least in part from disruptions in neural systems involving mPFC and limbic structures, in conjunction with insula cortex. Improved understanding of the contribution of these and other mechanisms should lead to better, more specific interventions for anxiety in ASD.

## Author Contributions

Both authors conceived the project. MS wrote the preliminary draft and both authors edited and approved the final manuscript.

## Conflict of Interest Statement

The authors declare that the research was conducted in the absence of any commercial or financial relationships that could be construed as a potential conflict of interest.
